# Higher TOX Genes Expression Is Associated With Poor Overall Survival for Patients With Acute Myeloid Leukemia

**DOI:** 10.3389/fonc.2021.740642

**Published:** 2021-10-08

**Authors:** Chaofeng Liang, Yujie Zhao, Cunte Chen, Shuxin Huang, Tairan Deng, Xiangbo Zeng, Jiaxiong Tan, Xianfeng Zha, Shaohua Chen, Yangqiu Li

**Affiliations:** ^1^ Key Laboratory for Regenerative Medicine of Ministry of Education, Institute of Hematology, School of Medicine, Jinan University, Guangzhou, China; ^2^ Department of Hematology, First Affiliated Hospital, Jinan University, Guangzhou, China; ^3^ Department of Clinical Laboratory, First Affiliated Hospital, Jinan University, Guangzhou, China

**Keywords:** TOX, prognosis, biomarker, immune checkpoint, acute myeloid leukemia

## Abstract

Thymocyte selection-associated HMG box (TOX) is a transcription factor that belongs to the high mobility group box (HMG-box) superfamily, which includes four subfamily members: TOX, TOX2, TOX3, and TOX4. TOX is related to the formation of multiple malignancies and contributes to CD8+ T cell exhaustion in solid tumors. However, little is known about the role of TOX genes in hematological malignancies. In this study, we explored the prognostic value of TOX genes from 40 patients with *de novo* acute myeloid leukemia (AML) by quantitative real-time PCR (qRT-PCR) in a training cohort and validated the results using transcriptome data from 167 *de novo* AML patients from the Cancer Genome Atlas (TCGA) database. In the training cohort, higher expression of *TOX* and *TOX4* was detected in the AML samples, whereas lower *TOX3* expression was found. Moreover, both the training and validation results indicated that higher *TOX2*, *TOX3*, and *TOX4* expression of AML patients (3-year OS: 0% *vs.* 37%, *P* = 0.036; 3-year OS: 4% *vs.* 61%, *P* < 0.001; 3-year OS: 0% *vs.* 32%, *P* = 0.010) and the AML patients with highly co-expressed *TOX*, *TOX2*, *TOX4* genes (3-year OS: 0% *vs.* 25% *vs.* 75%, *P* = 0.001) were associated with poor overall survival (OS). Interestingly, *TOX2* was positively correlated with *CTLA-4*, *PD-1*, *TIGIT*, and *PDL-2* (r_s_ = 0.43, *P* = 0.006; r_s_ = 0.43, *P* = 0.006; r_s_ = 0.56, *P* < 0.001; r_s_ = 0.54, *P* < 0.001). In conclusion, higher expression of TOX genes was associated with poor OS for AML patients, which was related to the up-regulation of immune checkpoint genes. These data might provide novel predictors for AML outcome and direction for further investigation of the possibility of using TOX genes in novel targeted therapies for AML.

## Introduction

In recent years with the improvement of chemotherapy regimens and the development of hematopoietic stem cell transplantation technology, acute myeloid leukemia (AML) patient treatment has achieved certain curative effects. However, there is still a high risk of relapse and a low disease-free survival rate (1, 2). The immune escape of tumor cells is a crucial cause of relapse and refractory AML (3). It has been shown that in the tumor microenvironment, tumor cells induce the expression of immune checkpoint (IC) genes, such as programmed cell death protein 1 (PD-1), cytotoxic T lymphocyte-associated molecule-4 (CTLA-4), and lymphocyte-activation gene 3 (LAG-3), leading to T cell exhaustion and immune escape (4–9). Clinical trials of targeted inhibitory antibodies, such as anti-PD-1 and anti-CTLA-4, in solid tumors have demonstrated their significant effects (10). In contrast, the clinical effectiveness of such immune therapies appears to be relatively different for different AML cases and clinical trials with different outcomes (11–13). Therefore, it is worth exploring the immune biomarkers that may be related to the effects of immune checkpoint blockade and revision of T cell exhaustion as well as their association with clinical outcome in AML (14).

Thymocyte selection-associated HMG box (TOX), a transcription factor that can bind to DNA, belongs to the high mobility group box (HMG-box) superfamily. TOX includes four subfamily members (TOX1-4, TOX1 is also known as TOX) (15). TOX is a crucial transcription factor related to the development of malignancies and contributing to CD8+ T cell exhaustion in patients with solid tumors (16–18). For example, TOX is positively correlated with larger tumor size, lower differentiation, later tumor node metastasis (TNM) stage, and facilitating endocytic recycling of PD-1 (17). In tumor-infiltrating CD8+ T cells from human melanoma and non-small cell lung cancer (NSCLC), increased expression of TOX in CD8+ T cells is associated with high expression of PD-1 (19). In contrast, there are few studies on TOX genes in hematological malignancies. TOX is highly expressed in acute lymphoblastic leukemia (ALL), particularly in T cell - ALL (T-ALL). High expression of TOX inhibits the function of the repair factors KU70/KU80 causing abnormal non-homologous end joining (NHEJ) repair (17). Although TOX is positively expressed in almost all ALL cases, *TOX* deletion has also been detected in ALL patients (20). Therefore, the mechanism by which TOX plays a role in ALL remains to be investigated.

In our previous study, we found higher TOX expression concurrent with PD-1, Tim-3, or CD244 in T cells from patients with B cell non-Hodgkin’s lymphoma (B-NHL), which suggested that TOX may be involved in inducing CD8+ T cell exhaustion by co-regulation with immune checkpoint proteins (21).

In this study, we investigated the expression characteristics and prognostic value of the TOX genes and analyzed the correlation between TOX and IC genes in peripheral blood (PB) samples from AML patients in our clinical center. The results were further validated with high-throughput sequencing data from The Cancer Genome Atlas (TCGA) database in a more significant number of patients.

## Materials and Methods

### PB Sample Information

**Figure 1:**
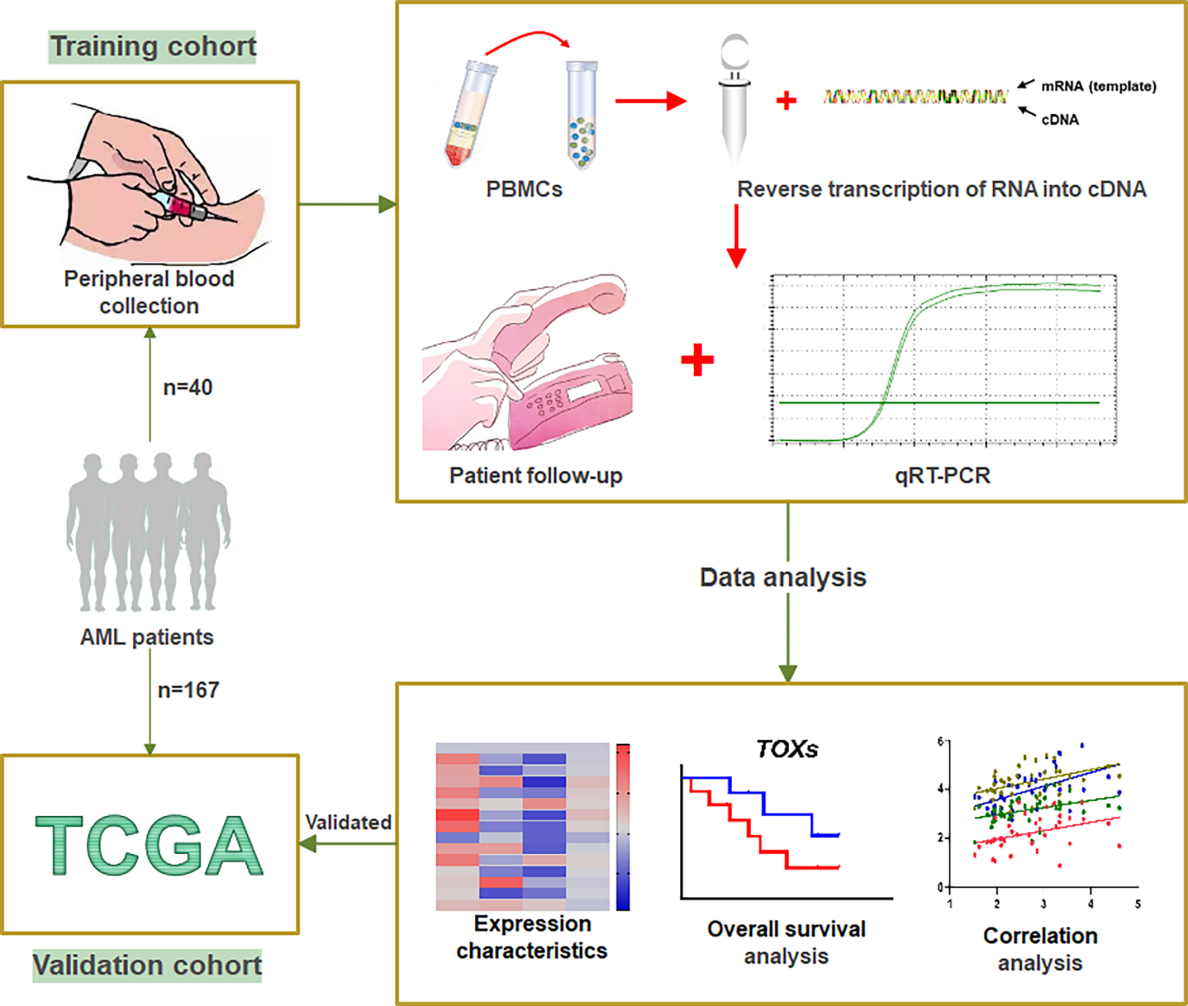
Workflow of study. A total of 40 AML patients from our clinical center were designated as the training cohort. Peripheral blood was collected from these patients to obtain PBMCs, which were used to translate RNA into cDNA. qRT-PCR was used to detect the expression levels of the TOX genes and *ICs*. After patient follow-up, the data were analyzed by expression characteristics, overall survival analysis, and correlation analysis. The gene expression data and clinical information of 167 *de novo* AML patients obtained from the TCGA were designated as a validation cohort. PBMCs, peripheral blood mononuclear cells; ICs, immune checkpoint genes; qRT-PCR, Quantitative Real-Time PCR; TCGA, The Cancer Genome Atlas.

In this study, we collected PB mononuclear cells from 40 *de novo* AML patients with informed consent (15 males and 25 females) who ranged in age from 12 to 83 years and provided informed consent from March 2016 to March 2021. We also included 17 AML-complete response (CR) patients (11 males and 6 females) whose ages ranged from 12 to 62 years (**Figure 1**). In addition, we collected PB white blood cells (WBCs) from 25 healthy individuals (HIs), including 12 males and 23 females, whose ages ranged from 19 to 70 years as a control population. Overall survival (OS) was defined as the time from diagnosis to death or last follow-up. The clinical information of the patients in the training cohort was listed in **Table 1**. This study was approved by the Ethics Committee of the School of Medicine of Jinan University [The ethical committee study number: (2015) Lun Shen Pi Ke No. 9].

**Table 1:** Clinical characteristics of AML patients.

Variables	Patients (total n = 40)
Age, mean ± SD, years	55 ± 19
Gender, n (%)	
Female	25 (62.5)
Male	15 (37.5)
WBC (x10^9^/L), mean ± SD	57.7 ± 100.8
BM blast cell, mean ± SD	70.0 ± 20.5
Risk stratification (ELN), n (%)	
Low	3 (7.5)
Intermediate	15 (37.5)
High	9 (22.5)
Unknown	13 (32.5)
Subtype, n (%)	
M2	11 (27.5)
M3	6 (15)
M4	3 (7.5)
M5	10 (25)
Unclassified	10 (25)
Gene mutation, n (%)	
FLT3	2 (5)
IDH2	2 (5)
NPM1	3 (7.5)
PML/RARA	6 (15)
RUNX1	2 (5)
WT1	4 (10)
No	4 (10)
Unknown	17 (42.5)
Cytogenetic abnormality, n (%)	
No	11 (27.5)
Yes	16 (40)
Unknown	13 (32.5)
Treatment, n (%)	
Chemotherapy	24 (60)
allo-HSCT	3 (7.5)
Other	13 (32.5)
Follow-up, median (range), days	316 (1-1608)
Status	
Alive	10 (25)
Dead	30 (75)

allo-HSCT, allogeneic hematopoietic stem cell transplantation; BM, bone marrow; ELN, European LeukmiaNet; SD, standard deviation; WBC, white blood cell.

### TCGA Dataset

The gene expression data and the clinical information of 167 *de novo* AML patients were obtained from the TCGA (https://cancergenome.nih.gov/) database by UCSC XENA (https://xenabrowser.net/datapages/) (6). The gene expression data from the TCGA database comprised the validation cohort for OS analysis and were used to validate the results of the training cohort.

### Quantitative Real-Time PCR

RNA isolation was performed using peripheral blood mononuclear cells (PBMCs) samples. Reverse transcription of RNA into cDNA was performed according to the manufacturer’s instructions for the Reverse Transcription Kit (ABI, USA). The gene expression levels were quantified according to the manufacturer’s instructions in the qRT-PCR kit (TIANGEN, China) (6), and *β2M* was used as an internal control. The sequences of the primers used for qRT-PCR are listed in **Supplementary Table 1**. The gene expression results are presented as the fold change lg (2^-ΔΔCT*100).

### Optimal Prognostic Cutoff Values

The Optimal prognostic cutoff values for *TOX*, *TOX2*, *TOX3*, and *TOX*4 were determined using the maximally selected rank statistics from the ‘maxstat’ R package, which was provided to the ‘survminer’ R package (22). This is an outcome-oriented method providing a value of a cut-point that corresponds to the most significant relationship with survival. According to the optimal cut-points of TOX genes, AML patients were divided into low- and high-expression groups to plot and compare Kaplan-Meier curves.

### Statistical Analysis

All statistical analyses were performed using Statistical Product and Service Solutions (SPSS) (version 22.0, IBM, Armonk, NY, USA), GraphPad Prism (version 8.4.2, CA, USA), and *R* (version 3.6.1, https://www.r-project.org/) as appropriate. Kaplan-Meier curves were plotted according to the optimal prognostic cutoff values (**Supplementary Figure S1**) for continuous variables, which were obtained using the “Survminer” package, and the log-rank test was used for comparison. A Correlation heatmap was generated using the “ggcorrplot” package, and it was analyzed using Spearman’s coefficient. Univariate and multivariate COX regression analyses were used to identify independent prognostic factors. The Mann-Whitney test was used for comparison between two groups, and the Kruskal-Wallis test was used to compare multiple gene expression groups. A two-tailed *p*-value < 0.05 was considered statistically significant.

## Results

### Expression Characteristics of TOX Genes in AML

**Figure 2:**
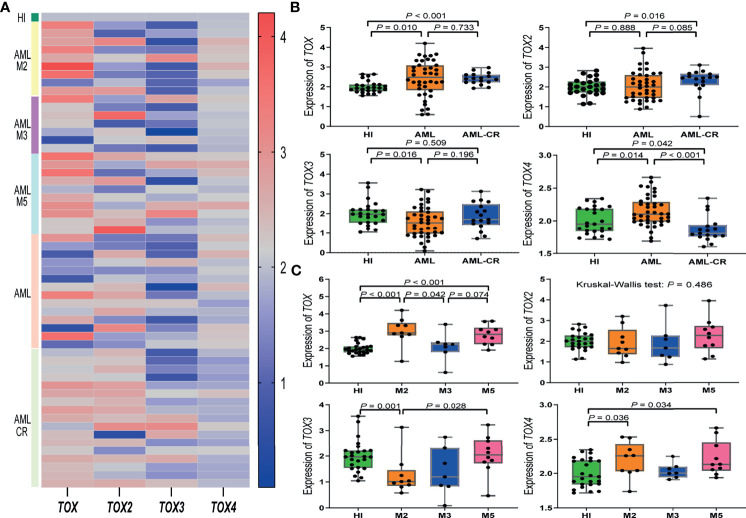
TOX gene expression levels in AML patients. **(A)** Heatmap of the expression levels of the TOX genes in AML patients from different subtypes and periods compared to a HI. **(B)** Expression levels of TOX genes in AML (orange) and AML-CR (blue) patients compared to HIs (green). **(C)** Expression levels of TOX genes in the M2 (orange), M3 (blue), and M5 (pink) subtypes in AML patients compared to HIs (green). CR, complete response; HI, healthy individuals.

The expression level of four TOX genes was characterized for 40 AML patients, 17 AML-CR patients, and 25 HIs (**Figure 2A**). Compared with the HIs, *TOX* was highly expressed in AML (median: 2.48 *vs.* 1.93, *P* = 0.010) and AML-CR (median: 2.40 *vs.* 1.93, *P* < 0.001) patients (**Figure 2B**). The expression of *TOX2* in the AML-CR group was higher than that in the HI group (median: 2.45 *vs.* 2.00, *P* = 0.016); however, the expression of *TOX3* was lower than that in the HI group (1.53 *vs.* 1.97, *P* = 0.016). The expression characteristics of *TOX4* was as follows: AML > HI > AML-CR (median: 2.10 *vs.* 1.95 *vs.* 1.81, AML *vs* AML-CR: *P* < 0.001; AML *vs* HI: *P* = 0.014; AML-CR *vs* HI: *P* = 0.042).

We further compared the expression level of the TOX genes in different AML subtypes in comparison to HIs (median: 1.93). The expression of *TOX* increased significantly in AML-M2 (median: 2.90, *P* < 0.001) and AML-M5 (median: 2.82, *P* < 0.001) patients. For *TOX*, the expression followed the pattern AML-M2 > AML-M5 > AML-M3 (AML-M2 *vs.* AML-M5: *P* = 0.388; AML-M3 *vs.* AML-M5: *P* = 0.074; AML-M2 *vs.* AML-M3: *P* = 0.042). There was no statistically significant difference for *TOX2* among the HI and AML subtypes (*P* = 0.486). *TOX3* expression in AML-M2 patients was lower than that in HIs (median: 1.01 *vs.* 1.96, *P* = 0.028). Interestingly, although *TOX3* was generally low in AML patients, its expression in AML-M5 patients (median: 2.04) was significantly higher than that in AML-M2 patients, and it had the following expression pattern: AML-M5 > AML-M3 > AML-M2 (AML-M5 *vs.* AML-M3: *P* = 0.193; AML-M3 *vs.* AML-M2: *P* = 0.837; AML-M5 *vs.* AML-M2: *P* = 0.028). The expression of *TOX4* in AML-M2 (median: 2.25) and AML-M5 (median: 2.13) patients maintained an upward trend compared with the HI group (median: 1.95, *P* = 0.036, *P* = 0.034); however, there was no statistically significant difference between the AML-M3 and HI groups (**Figure 2C**).

### Higher Expression of TOX Genes Is Associated With Poor OS in AML Patients

**Figure 3:**
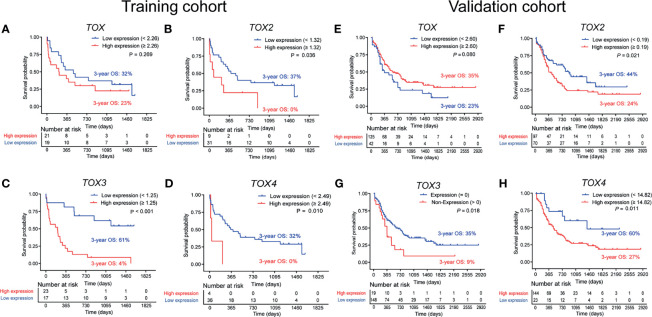
Overall survival (OS) analysis of *TOX*, *TOX2*, *TOX3*, and *TOX4* from training **(A–D)** and validation **(E–H)** cohort. According to optimal cutoff values, the TOX genes were divided into High expression (red line) and Low expression (blue line) groups, which were plotted in Kaplan-Meier curves (top) with the number at risk AML patients (bottom).

To investigate the role of altered *TOX* expression in the clinical outcome of AML patients, we collected the clinical information of the AML patients and analyzed the association between the TOX expression level and the OS of AML patients by Kaplan-Meier curves. The optimal prognostic cutoff value for *TOX*, *TOX2*, *TOX3*, and *TOX4* was 2.26, 1.32, 1.25, and 2.49, respectively (**Supplementary Figures S1A–D**). Using these values, we divided the patients into high and low expression groups (**Figures 3A–H**). The results demonstrated that AML patients with high *TOX* expression were associated with short survival time and poor OS in the training cohort, but there was no statistically significant difference (3-year OS 23% *vs.* 32%, *P* = 0.269, **Figure 3A**). The 3-year restricted mean survival time (RMST) of the high expression group was 397 days, and the 3-year RMST of the low expression group was 578 days (**Supplementary Figure S2A**). Similarly, *TOX* expression in the TCGA data had no statistically significant difference (*P* = 0.080, **Figure 3E**). In the training cohort, AML patients with high *TOX2*, *TOX3*, and *TOX4* expression had shorter survival time and more inferior OS (3-year OS: 0% *vs.* 37%, *P* = 0.036; 3-year OS: 4% *vs.* 61%, *P* < 0.001; 3-year OS: 0% *vs.* 32%, *P* = 0.010, **Figures 3B–D**). The 3-year RMST of the high expression group was 412, 270, 67 days, respectively, and the 3-year RMST of the low expression group was 727, 776, 531 days, respectively (**Supplementary Figures S2B–D**). These findings were confirmed in the validation cohort (3-year OS: 24% *vs.* 44%, *P* = 0.021; 3-year OS: 9% *vs.* 35%, *P* = 0.018; 3-year OS: 27% *vs.* 60%, *P* = 0.011, **Figures 3F–H**).

**Figure 4:**
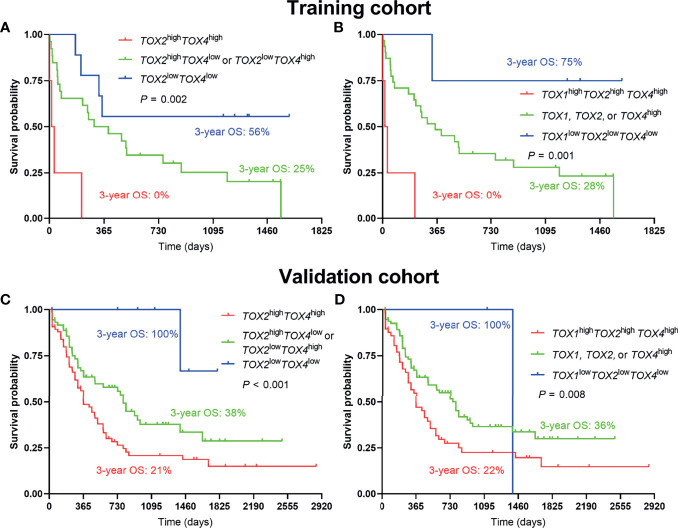
Co-expression of TOX genes in predicting poor OS of AML patients. Kaplan-Meier curves are shown for co-high expression, single high expression, and co-low expression of *TOX2*/*TOX4* **(A)** and *TOX*/*TOX2*/*TOX4* **(B)** in training and validation cohort **(C, D)**.

Considering an additive effect on the outcome if multiple TOX genes are aberrantly elevated, we characterize the predictive value of co-expression of TOX genes in AML. Using the co-expression of TOX genes to evaluate the OS, we found that lower OS was observed in *TOX2* ^high^ *TOX4* ^high^ AML patients in comparison with *TOX2* ^high^ *TOX4* ^low^ or *TOX2* ^low^ *TOX4* ^high^ AML patients and *TOX2* ^low^ *TOX4* ^low^ AML patients (3-year OS: 0% *vs.* 25% *vs.* 56%, *P* = 0.002, **Figure 4A**). In addition, *TOX* ^high^ *TOX2* ^high^ *TOX4* ^high^ AML patients are also related to the poor prognosis of patients (3-year OS: 0% *vs.* 25% *vs.* 75%, *P* = 0.001, **Figure 4B**). The same results were also confirmed in the validation cohort (3-year OS: 21% *vs.* 38% vs. 100%, *P* < 0.001; 3-year OS: 22% *vs.* 36% *vs.* 100%, *P* = 0.008, **Figures 4C, D**).

To better understand the relationship between *TOX*, *TOX2*, *TOX3*, and *TOX4* and other impact factors, COX regression analysis was used. When age, gender, AML subtype, ELN risk group, CBF rearrangements, hematologic parameters, treatment, *TOX*, *TOX2*, *TOX3*, and *TOX4* were included in univariate COX regression analysis, only age, *TOX2*, *TOX3*, and *TOX4* were significantly associated with poor overall survival in AML patients. Therefore, age was used for adjusting *TOX2*, *TOX3*, and *TOX4* in AML patients. Importantly, we found that high expression of *TOX2*, *TOX3*, and *TOX4* is an independent factor affecting survival. Compared with patients with low expression of *TOX2*, *TOX3*, and *TOX4*, those with high expression are at higher risk of death than those with low expression: *TOX2*: *P* = 0.005, hazard ratio (HR) = 1.03 (95% confidence interval (CI): 1.01-1.05); *TOX3*: *P* = 0.037, HR = 1.02 (95% CI: 1.00-1.04); *TOX4*: *P* = 0.032, HR = 1.03 (95% CI: 1.00-1.05). However, in the univariate COX regression model, the expression level of *TOX* was not significantly associated with the OS of AML patients (HR = 1.60, 95% CI: 0.77-3.35, *P* = 0.210, **Table 2**).

**Table 2:** Uni- and multivariate regression analysis of *TOX2*, *TOX3*, and *TOX4* in AML patients.

Variables			Multivariate regression					
	Univariate regression		*TOX2*/Age		*TOX3*/Age		*TOX4*/Age	
	HR (95% CI)	*P* value	HR (95% CI)	*P* value	HR (95% CI)	*P* value	HR (95% CI)	*P* value
Gender								
Female	Reference							
Male	0.68 (0.32, 1.45)	0.319						
Age, years	1.03 (1.01, 1.05)	0.007	1.03 (1.01, 1.05)	0.005	1.02 (1.00, 1.04)	0.037	1.03 (1.00, 1.05)	0.032
Subtype								
M2	Reference							
M3	0.15 (0.02, 1.12)	0.075						
M5	1.91 (0.69, 5.25)	0.212						
WBC, 10^9^/L	1.00 (1.00, 1.00)	0.888						
BM blast cell, %	0.99 (0.97, 1.01)	0.402						
Risk stratification (ELN)								
Low	Reference							
Intermediate	2.17 (0.48, 9.79)	0.314						
High	1.38 (0.27, 7.21)	0.700						
Treatment								
allo-HSCT	Reference							
Chemotherapy	4.23 (0.56, 31.98)	0.162						
Other	4.47 (0.57, 34.86)	0.153						
*TOX*								
Low expression	Reference							
High expression	1.60 (0.77, 3.35)	0.210						
*TOX2*								
Low expression	Reference		Reference					
High expression	2.98 (1.03, 8.59)	0.043	3.22 (1.10, 9.49)	0.034				
*TOX3*								
Low expression	Reference				Reference			
High expression	4.47 (1.89, 10.59)	0.001			3.92 (1.61, 9.58)	0.003		
*TOX4*								
Low expression	Reference						Reference	
High expression	5.79 (1.83, 18.35)	0.003					3.50 (1.04, 11.72)	0.043

allo-HSCT, allogeneic hematopoietic stem cell transplantation; BM, bone marrow; CI, confidence interval; ELN, European LeukmiaNet; HR, hazard ratio; WBC, white blood cell.

### Correlation of TOX and IC Genes Expression in AML

**Figure 5:**
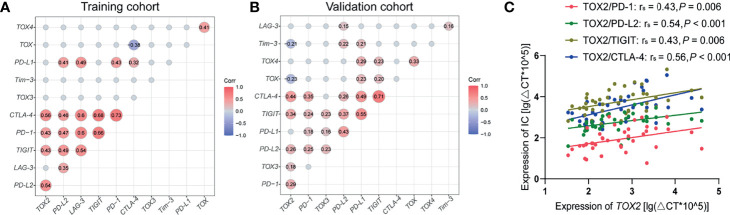
Correlation between TOX and IC genes in peripheral blood from AML patients. **(A)** Correlation matrix heatmap showing the relationship between TOX and IC genes from training **(A)** and validation **(B)** cohort. Correlated genes (*P* < 0.05) are displayed in red (positive correlation) or blue (negative correlation), and the degree of correlation is represented by the number in the middle of the circle and the shade of the color. **(B)** Correlations between the expression levels of *TOX2* and that of *PD-1* (red), *PD-L2* (green), *TIGIT* (brown), and *CTLA-4* (blue) from training cohort **(C)**. IC, immune checkpoint.

Based on the previous finding of TOX expression concurrent with that of PD-1 and Tim-3 in T cells from patients with lymphoma (21), we analyzed the correlation of the gene expression level of the TOX genes and IC genes in AML patients (**Figures 5A–C**). Significantly, *TOX2* has a positive correlation with *TIGIT*, *PD-1*, *CTLA-4*, and *PDL2* (r_s_ = 0.43, *P* = 0.006; r_s_ = 0.43, *P* = 0.006; r_s_ = 0.56, *P* < 0.001; r_s_ = 0.54, *P* < 0.001). Moreover, the expression levels of *TOX* and *TOX4* had a positive correlation (r_s_ = 0.41, *P* = 0.008), while the expression level of *TOX* and *TOX2* demonstrated a trend toward a negative correlation (r_s_ = -0.133, *P* = 0.412). Interestingly, there was a significantly negative correlation between *TOX* and *TOX2* expression in the TCGA dataset (r_s_ = -0.23, *P* = 0.003). These results were confirmed in the validation cohort (r_s_ = 0. 34, *P* < 0.001; r_s_ = 0.29, *P* < 0.001; r_s_ = 0. 44, *P* < 0.001; r_s_ = 0. 26, *P* < 0.001, **Figure 5B**).

## Discussion

The biomarkers for AML outcome, particularly those related to immune suppression, which often occurs in cancer immune escape, are far from clear. Recent studies have shown that TOX is a crucial transcription factor that contributes to T cell exhaustion and is involved in tumor development (16, 23). However, how TOX is altered in AML remains unclear. In this study, we explored the expression characteristics of the four TOX family members in AML samples. Interestingly, the expression patterns of the TOX genes were different with the expression of *TOX* and *TOX4* significantly increased, *TOX3* was decreased, and *TOX2* demonstrated an increasing expression trend. These differences may be due to the functional differences of TOXs in the AML subtypes and may be involved in clinical outcome. It is well known that AML includes eight subtypes and is a heterogeneous disease (24). For instance, AML-M3 is a particular subtype with a favorable outcome. Indeed, the expression of TOX genes in patients of different AML subtypes i.e., AML-M2, M3, and M5, was different. Overall, *TOX* and *TOX4* had low expression in the M3 group and high expression in the M2 and M5 groups, while *TOX3* was low in AML-M2 and high in AML-M5. Therefore, the study of the TOX family as a biomarker may provide particular predictive value for the study of patients with different types.

It has been reported that both *TOX* and *TOX2* are correlated with CD8+ T cell exhaustion (25). In this study, we also found that *TOX2* is positively correlated with *CTLA-4*, *PD-1*, *TIGIT*, and *PDL-2* in AML samples from either our center or from the TCGA dataset. Previous studies have indicated that higher *PD-1, PD-L1*, or *CTLA-4* expression is associated with poor OS in AML (6). Thus, *TOX2* may be one more biomarker for predicting the T cell immune suppression related to the clinical outcome of AML. However, we did not find any association between the expression of *TOX* and the immune checkpoint genes. The reason for this discrepancy may be the level of *TOX* expression in different cells. It is possible that the association between TOX and PD-1 or CTLA-4 co-expression only occurs in T cells (21) and not PBMCs, which include a high percentage of AML cells. We found that TOX genes are also expressed in AML cell lines and primary AML cells (data not shown), which suggests that there are different patterns of expression for TOX genes in T cells and AML cells, which may play a different role. *TOX3* is an essential protective transactivator in neurons (26) and plays different roles in various tumors (27–29). Moreover, *TOX4* regulates the cell cycle and fate (30, 31), but there is no information regarding the expression characteristics of *TOX4* in cancer or hematological malignancies. In this study, we characterized the expression patterns of *TOX3* and *TOX4* in AML. Interestingly, the expression of *TOX3* was significantly decreased and different from the other TOX genes, while *TOX4* was highly expressed. Whether these genes play different regulatory roles in AML requires further investigation. We also considered whether there is any correlation or complementation between the expression of the TOX genes. From our results, we could find a positive correlation between *TOX* and *TOX4* in the training and validation groups, while a negative correlation or correlation trend between *TOX* and *TOX2* expression was found. Whether this is complementary regulation remains an open question.

To further discuss the role of the altered expression of the TOX genes in AML, we explored the association between the expression of the TOX genes and the OS of AML patients. Our results demonstrated that AML patients with high *TOX2* expression have an inferior OS. Combined with the finding that *TOX2* positively correlates with IC genes and is highly expressed in AML-CR patients, overexpression of *TOX2* together with T cell exhaustion may be a reason why AML patients have poor OS. It is worth further investigating the value in predicting OS for AML by evaluating the co-expression of *TOX* with immune checkpoint genes (32). Although the expression of *TOX3* is decreased in AML, patients with higher *TOX3* expression have an inferior 3-year OS, the function of *TOX3* is needed to further characterize. Moreover, we found that patients with increased *TOX3* expression are primarily AML-M5 patients. These findings may provide a precise and valuable predictor of OS for AML. Similarly, higher expression of *TOX4* is also related to poor prognosis. An interesting finding is that when the *TOX*, *TOX2*, *TOX4* genes co-expressed highly in AML patients, the prognosis of these patients is significantly poor. This finding indicates that TOX genes play a negative role in AML patients to a large extent. Overall, TOX genes may be potential biomarkers for predicting clinical outcomes in AML, and their blockade may be considered a new direction for the treatment of AML patients.

In summary, in this study, we characterized the altered expression of TOX genes in AML and defined their different roles. We also demonstrated that *TOX2* is positively correlated with the *CTLA-4*, *PD-1*, *TIGIT*, and *PDL-2* genes. Moreover, higher expression of *TOX2*, *TOX3*, and *TOX4* of AML patients and the AML patients with highly co-expressed *TOX*, *TOX2*, *TOX4* genes were associated with poor OS for AML patients, which may be related to the upregulation of immune checkpoint genes. These data indicate that TOX genes may be novel predictors for clinical outcomes in AML. Moreover, TOX, as the upstream molecule of immune checkpoint proteins, is not only expressed in AML cells but also associated with T cell exhaustion, which might provide direction for future investigations of the possibility of the dual effect of TOX targeted inhibition, inhibiting proliferation of AML cells and revising T cell exhaustion and restoring anti-AML T cell function.

## Data Availability Statement

The original contributions presented in the study are included in the article/**Supplementary Material**. Further inquiries can be directed to the corresponding authors.

## Ethics Statement

The studies involving human participants were reviewed and approved by Ethics Committee of the School of Medicine of Jinan University. Written informed consent to participate in this study was provided by the participants’ legal guardian/next of kin.

## Author Contributions

SC and YL contributed to the concept development and study design and edited the manuscript. CL performed the experiments, interpreted the data, and wrote the manuscript. YZ and CC helped write the manuscript. SH, TD, and XBZ supported reviewing the references and preparing figures. JT and XFZ collected the clinical information and provided clinical peripheral blood samples. All authors contributed to the article and approved the submitted version.

## Funding

This work was supported by grants from the National Natural Science Foundation of China (Nos. 82070152, 81770152, and 81570143), the Guangzhou Science and Technology Project (Nos. 201807010004 and 201803040017), and the Training Program of Innovation and Entrepreneurship for Undergraduates of Jinan University (No. CX20150).

## Conflict of Interest

The authors declare that the research was conducted in the absence of any commercial or financial relationships that could be construed as a potential conflict of interest.

## Publisher’s Note

All claims expressed in this article are solely those of the authors and do not necessarily represent those of their affiliated organizations, or those of the publisher, the editors and the reviewers. Any product that may be evaluated in this article, or claim that may be made by its manufacturer, is not guaranteed or endorsed by the publisher.

## Supplementary Material

Supplementary Table 1 Primers for qRT-PCR.

Supplementary Figure 1 Optimal cutoff values for *TOX*, *TOX2*, *TOX3*, and *TOX4* from training **(A–D)** and validation **(E–H)** cohort.

Supplementary Figure 2 Relationship between RMST and the expression levels of AML patients. RMST, restricted mean survival time.

## Abbreviations

AML, acute myeloid leukemia; allo-HSCT, allogeneic hematopoietic stem cell transplantation; B-NHL, B-non-Hodgkin’s lymphoma; BM, bone marrow; CI, confidence interval; CR, complete response; CTLA-4, cytotoxic T lymphocyte-associated molecule-4; ELN, European LeukmiaNet, HI, healthy individuals; HMG-box, high mobility group box; HR, hazard ratio; ICs, immune checkpoint genes; LAG-3, lymphocyte-activation gene 3; NHEJ, non-homologous end joining; NSCLC, non-small cell lung cancer; OS, overall survival; PB, peripheral blood; PBMCs, peripheral blood mononuclear cells; PD-1, programmed cell death protein 1; qRT-PCR, quantitative real-time PCR; RMST, restricted mean survival time; SD, standard deviation; SPSS, Statistical Product and Service Solutions; T-ALL, T cell - acute lymphoblastic leukemia; TCGA, The Cancer Genome Atlas; TNM, tumor node metastasis; TOX, thymocyte selection-associated HMG box; WBC, white blood cell.

## References

[1] Döhner H Weisdorf DJ Bloomfield CD. Acute Myeloid Leukemia. N Engl J Med (2015) 373(12):1136–52. doi: 10.1056/NEJMra1406184 26376137

[2] Stein EM Tallman MS. Emerging Therapeutic Drugs for AML. Blood (2016) 127(1):71–8. doi: 10.1182/blood-2015-07-604538 PMC4915807 26660428

[3] Mougiakakos D. The Induction of a Permissive Environment to Promote T Cell Immune Evasion in Acute Myeloid Leukemia: The Metabolic Perspective. Front Oncol (2019) 9:1166. doi: 10.3389/fonc.2019.01166 31781489 PMC6851227

[4] Kursunel MA Esendagli G. A Co-Inhibitory Alliance in Myeloid Leukemia: TIM-3/Galectin-9 Complex as a New Target for Checkpoint Blockade Therapy. EBioMedicine (2017) 23:6–7. doi: 10.1016/j.ebiom.2017.08.002 28801238 PMC5605328

[5] Kasakovski D Xu L Li Y. T Cell Senescence and CAR-T Cell Exhaustion in Hematological Malignancies. J Hematol Oncol (2018) 11(1):91. doi: 10.1186/s13045-018-0629-x 29973238 PMC6032767

[6] Chen C Liang C Wang S Chio CL Zhang Y Zeng C. Expression Patterns of Immune Checkpoints in Acute Myeloid Leukemia. J Hematol Oncol (2020) 13(1):28. doi: 10.1186/s13045-020-00853-x 32245463 PMC7118887

[7] Yasinska IM Sakhnevych SS Pavlova L Teo Hansen Selnø A Teuscher Abeleira AM Benlaouer O. The Tim-3-Galectin-9 Pathway and Its Regulatory Mechanisms in Human Breast Cancer. Front Immunol (2019) 10:1594. doi: 10.3389/fimmu.2019.01594 31354733 PMC6637653

[8] Martínez-López M Soto M Iborra S Sancho D. Leishmania Hijacks Myeloid Cells for Immune Escape. Front Microbiol (2018) 9:883. doi: 10.3389/fmicb.2018.00883 29867798 PMC5949370

[9] Chen C Zeng C Li Y. The Importance of Genomic Predictors for Clinical Outcome of Hematological Malignancies. Blood Sci (2021) 3(3):93–5. doi: 10.1097/bs9.0000000000000075 PMC8974908 35402837

[10] Wang J Yang T Xu J. Therapeutic Development of Immune Checkpoint Inhibitors. Adv Exp Med Biol (2020) 1248:619–49. doi: 10.1007/978-981-15-3266-5_23 32185726

[11] Gale RP. Will Immune Therapy Cure Acute Myeloid Leukemia? Blood Sci (2019) 1(1):2–3. doi: 10.1097/bs9.0000000000000024 PMC8974985 35402798

[12] Zhang X Yang Y Fan D Xiong D. The Development of Bispecific Antibodies and Their Applications in Tumor Immune Escape. Exp Hematol Oncol (2017) 6:12. doi: 10.1186/s40164-017-0072-7 28469973 PMC5414286

[13] Zhu R Tao H Lin W Tang L Hu Y. Identification of an Immune-Related Gene Signature Based on Immunogenomic Landscape Analysis to Predict the Prognosis of Adult Acute Myeloid Leukemia Patients. Front Oncol (2020) 10:574939. doi: 10.3389/fonc.2020.574939 33330048 PMC7714942

[14] Salik B Smyth MJ Nakamura K. Targeting Immune Checkpoints in Hematological Malignancies. J Hematol Oncol (2020) 13(1):111. doi: 10.1186/s13045-020-00947-6 32787882 PMC7425174

[15] O'Flaherty E Kaye J. TOX Defines a Conserved Subfamily of HMG-Box Proteins. BMC Genomics (2003) 4(1):13. doi: 10.1186/1471-2164-4-13 12697058 PMC155677

[16] Liang C Huang S Zhao Y Chen S Li Y. TOX as a Potential Target for Immunotherapy in Lymphocytic Malignancies. Biomark Res (2021) 9(1):20. doi: 10.1186/s40364-021-00275-y 33743809 PMC7981945

[17] Lobbardi R Pinder J Martinez-Pastor B Theodorou M Blackburn JS Abraham BJ. TOX Regulates Growth, DNA Repair, and Genomic Instability in T-Cell Acute Lymphoblastic Leukemia. Cancer Discov (2017) 7(11):1336–53. doi: 10.1158/2159-8290.Cd-17-0267 PMC5683427 28974511

[18] Dulmage BO Akilov O Vu JR Falo LD Geskin LJ. Dysregulation of the TOX-RUNX3 Pathway in Cutaneous T-Cell Lymphoma. Oncotarget (2019) 10(33):3104–13. doi: 10.18632/oncotarget.5742 PMC6517103 31139323

[19] Kim K Park S Park SY Kim G Park SM Cho JW. Single-Cell Transcriptome Analysis Reveals TOX as a Promoting Factor for T Cell Exhaustion and a Predictor for Anti-PD-1 Responses in Human Cancer. Genome Med (2020) 12(1):22. doi: 10.1186/s13073-020-00722-9 32111241 PMC7048139

[20] Bardet V Couque N Cattolico L Hetet G Devaux I Duprat S. Molecular Analysis of Nonrandom 8q12 Deletions in Acute Lymphoblastic Leukemia: Identification of Two Candidate Genes. Genes Chromosomes Cancer (2002) 33(2):178–87. doi: 10.1002/gcc.10014 11793444

[21] Huang S Liang C Zhao Y Deng T Tan J Lu Y. Increased TOX Expression Concurrent With PD-1, Tim-3, and CD244 in T Cells From Patients With Non-Hodgkin Lymphoma. Asia Pac J Clin Oncol (2021). doi: 10.1111/ajco.13545 33608984

[22] Laska E Meisner M Wanderling J. A Maximally Selected Test of Symmetry About Zero. Stat Med (2012) 31(26):3178–91. doi: 10.1002/sim.5384 22729950

[23] Guo R Lü M Cao F Wu G Gao F Pang H. Single-Cell Map of Diverse Immune Phenotypes in the Acute Myeloid Leukemia Microenvironment. biomark Res (2021) 9(1):15. doi: 10.1186/s40364-021-00265-0 33648605 PMC7919996

[24] Chen C Chio CL Zeng H Li Y. High Expression of CD56 May Be Associated With Favorable Overall Survival in Intermediate-Risk Acute Myeloid Leukemia. Hematol (Amsterdam Netherlands) (2021) 26(1):210–4. doi: 10.1080/16078454.2021.1880734 33594945

[25] Seo H Chen J González-Avalos E Samaniego-Castruita D Das A Wang YH. TOX and TOX2 Transcription Factors Cooperate With NR4A Transcription Factors to Impose CD8(+) T Cell Exhaustion. Proc Natl Acad Sci USA (2019) 116(25):12410–5. doi: 10.1073/pnas.1905675116 PMC6589758 31152140

[26] Yuan SH Qiu Z Ghosh A. TOX3 Regulates Calcium-Dependent Transcription in Neurons. Proc Natl Acad Sci USA (2009) 106(8):2909–14. doi: 10.1073/pnas.0805555106 PMC2650364 19196971

[27] Han YJ Zhang J Zheng Y Huo D Olopade OI. Genetic and Epigenetic Regulation of TOX3 Expression in Breast Cancer. PloS One (2016) 11(11):e0165559. doi: 10.1371/journal.pone.0165559 27806084 PMC5091860

[28] Zeng D Lin H Cui J Liang W. TOX3 is a Favorable Prognostic Indicator and Potential Immunomodulatory Factor in Lung Adenocarcinoma. Oncol Lett (2019) 18(4):4144–52. doi: 10.3892/ol.2019.10748 PMC6732997 31516613

[29] He Y Liu H Chen Q Shao Y Luo S. Relationships Between SNPs and Prognosis of Breast Cancer and Pathogenic Mechanism. Mol Genet genomic Med (2019) 7(9):e871. doi: 10.1002/mgg3.871 31317673 PMC6732281

[30] Lee JH You J Dobrota E Skalnik DG. Identification and Characterization of a Novel Human PP1 Phosphatase Complex. J Biol Chem (2010) 285(32):24466–76. doi: 10.1074/jbc.M110.109801 PMC2915683 20516061

[31] Vanheer L Song J De Geest N Janiszewski A Talon I Provenzano C. Tox4 Modulates Cell Fate Reprogramming. J Cell Sci (2019) 132(20):jcs232223. doi: 10.1242/jcs.232223 31519808 PMC6826012

[32] Chen C Xu L Gao R Wang S Zhang Y Wang C. Transcriptome-Based Co-Expression of BRD4 and PD-1/PD-L1 Predicts Poor Overall Survival in Patients With Acute Myeloid Leukemia. Front Pharmacol (2020) 11:582955. doi: 10.3389/fphar.2020.582955 33658927 PMC7917577

