# Supplementary material for: Higher TOX Genes Expression Is Associated With Poor Overall Survival for Patients With Acute Myeloid Leukemia
Source: Front Oncol. 2021 Oct 8;11:740642. doi: 10.3389/fonc.2021.740642 (PMC8532529; doi:10.3389/fonc.2021.740642)
Supplement: Supplementary Table 1 — Primers for qRT-PCR. [file Table_1.docx]

**Supplementary Table 1** Primers for qRT-PCR

| Target | Sequence |
| --- | --- |
| *β2M*-F | 5′-TACACTGAATTCACCCCCAC-3′ |
| *β2M*-R | 5′-CATCCAATCCAAATGCGGCA-3′ |
| *PD-1*-F | 5′-CTCAGGGTGACAGAGAGAAG-3′ |
| *PD-1*- R | 5′-GACACCAACCACCAGGGTTT-3′ |
| *PDL1*-F | 5′-TATGGTGGTGCCGACTACAA-3′ |
| *PDL1*-R | 5′-TGCTTGTCCAGATGACTTCG-3′ |
| *PDL2*-F | 5′-ACAGTGCTATCTGAACCTGTGG-3′ |
| *PDL2*-R | 5′-CTGCAGGCCACCGAATTCTT-3′ |
| *Tim-3*-F | 5′-TCCAAGGATGCTTACCACCAG-3' |
| *Tim-3*-R | 5′-GCCAATGTGGATATTTGTGTTAGATT-3' |
| *LAG3*-F | 5′-CTAGCCCAGGTGCCCAACGC-3′ |
| *LAG3*-R | 5′-GCCTGCGGAGGGTGAATCCC-3′ |
| *CTLA*-4-F | 5′-GCCCTGCACTCTCCTGTTTTT-3′ |
| *CTLA*-4-R | 5′-GGTTGCCGCACAGACTTCA-3′ |
| *TIGIT*-F | 5′-TCTGCATCTATCACACCTACCC-3′ |
| *TIGIT*-R | 5′-CCACCACGATGACTGCTGT-3′ |
| *TOX*-F | 5′-GCACCTGAATGAAGTTGAGTCT-3′ |
| *TOX*-R | 5′-CCTGGCC-CAGCATATTGGAG-3′ |
| *TOX2*-F | 5′-AGTCGGAAGTGCATTTCAAGAT-3′ |
| *TOX2*-R | 5′-GGCCTGAGTGTCTCTGAAGA-3′ |
| *TOX3*-F | 5′-TATGCCTCACACATCTCCTTCA-3′ |
| *TOX3*-R | 5′-ATGGCTCTGTTGGCTTCATC-3′ |
| *TOX4*-F | 5′-TGACAATTACCTGACGATCACAG-3′ |
| *TOX4*-R | 5′-TCCAAGGAGATAGGTGGGATTTC-3′ |
