# Supplementary figures and images for: Higher TOX Genes Expression Is Associated With Poor Overall Survival for Patients With Acute Myeloid Leukemia
Source: Front Oncol. 2021 Oct 8;11:740642. doi: 10.3389/fonc.2021.740642 (PMC8532529; doi:10.3389/fonc.2021.740642)

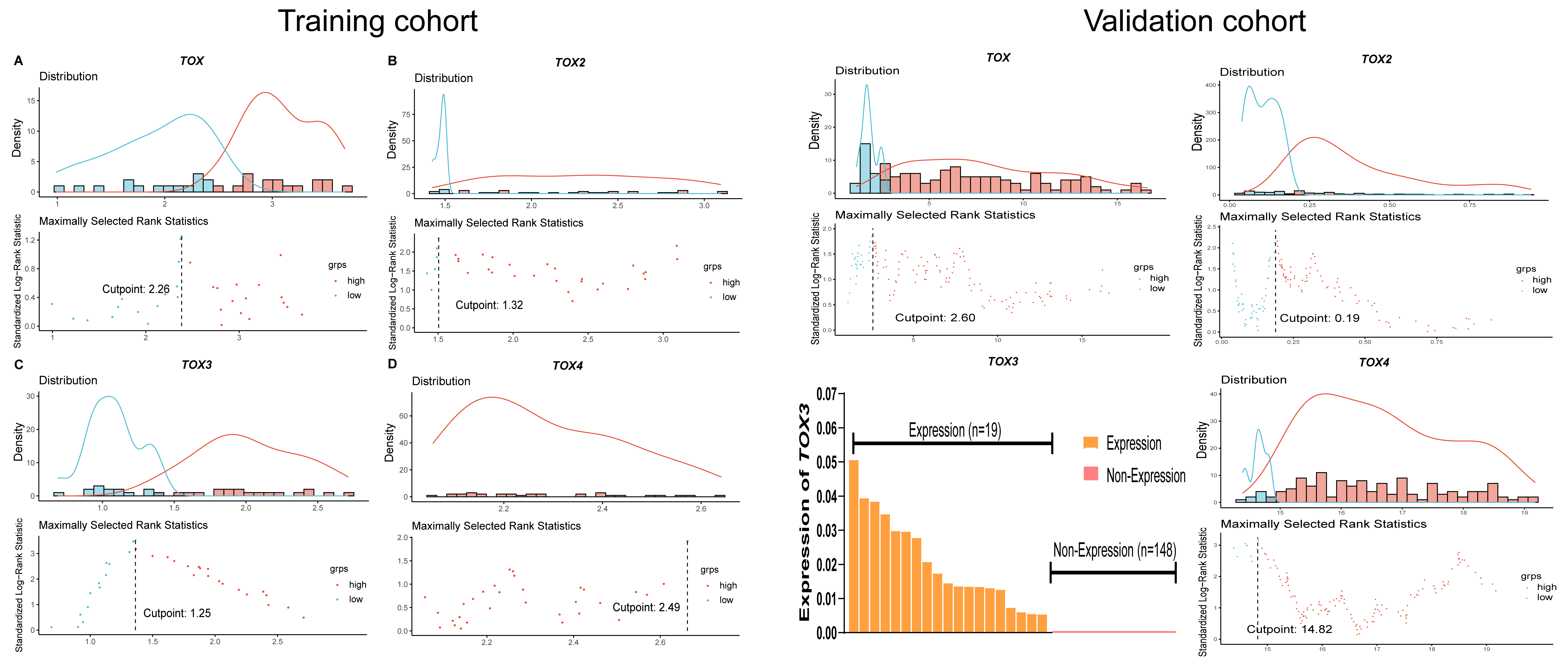

Supplement: Supplementary Figure 1 — Optimal cutoff values for TOX, TOX2, TOX3, and TOX4 from training (A–D) and validation (E–H) cohort. [file Image_1.tif]

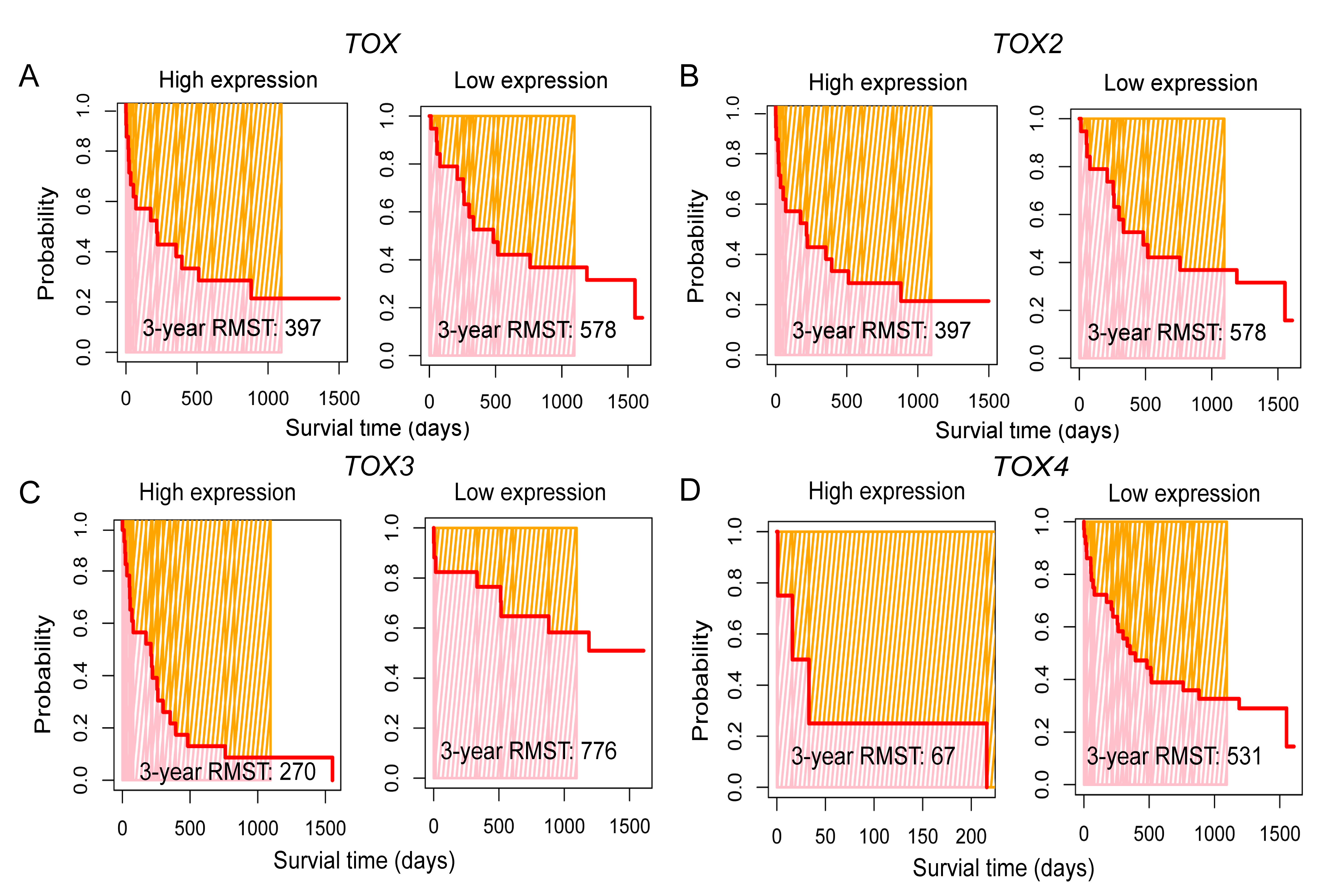

Supplement: Supplementary Figure 2 — Relationship between RMST and the expression levels of AML patients. RMST, restricted mean survival time. [file Image_2.tif]
